# 3.1 ENHANCED PARVALBUMIN NETWORK ACTIVITY PROLONGS CRITICAL PERIOD PLASTICITY

**DOI:** 10.1093/schbul/sby014.003

**Published:** 2018-04-01

**Authors:** Hensch Takao

**Affiliations:** Harvard University

## Abstract

**Background:**

Oscillations in neuronal activity tie the pathophysiology of schizophrenia to alterations in local processing and large-scale coordination, and these alterations in turn can lead to the cognitive and perceptual disturbances observed in schizophrenia. Here, we focus on the dual role of fast-spiking, parvalbumin (PV+) networks in the generation of gamma oscillations and critical periods of brain plasticity.

**Methods:**

We generated a mouse model of reduced recurrent inhibition only within local PV+ cell networks by selective removal of GABAA receptor alpha1 subunits (PV-α1 KO mice). Electroencephalography (EEG), PV+ immunohistochemistry, perineuronal net (PNN) labeling and redox balance were compared to cortical measures of brain plasticity (loss of visual acuity, formation of preference behaviors) that are typically limited to a critical period early in life.

**Results:**

PV-α1 KO mice exhibit chronically enhanced gamma-oscillations and extended juvenile forms of cortical plasticity into adulthood. Acute pharmacological suppression of excitatory input restored E-I balance onto these disinhibited PV+ cells and returned baseline EEG power to normal levels, preventing the extended plasticity. Enhanced gamma oscillations were further found to compromise the integrity of perineuronal nets (PNNs) surrounding PV+ cells, elevating oxidative stress and the turnover of metallopeptidases and structural components of the PNN. All of these aspects were also reversed by pharmacological dampening of excitation onto PV+ cells.

**Discussion:**

Cortical gamma oscillations are associated with plasticity and cognition. Our results provide a cellular explanation of how elevated gamma oscillations may promote ectopic brain plasticity by regulating the extracellular matrix which normally stabilizes cortical circuitry. These results carry broad implications for subjects at-risk for schizophrenia who exhibit heightened gamma oscillations prior to psychosis onset (see talk by P Uhlhaas).

